# Complex-Exponential-Based Bio-Inspired Neuron Model Implementation in FPGA Using Xilinx System Generator and Vivado Design Suite

**DOI:** 10.3390/biomimetics8080621

**Published:** 2023-12-18

**Authors:** Maruf Ahmad, Lei Zhang, Kelvin Tsun Wai Ng, Muhammad E. H. Chowdhury

**Affiliations:** 1 Faculty of Engineering and Applied Science, University of Regina, Regina, SK S4S 0A2, Canada; mah370@uregina.ca (M.A.); kelvin.ng@uregina.ca (K.T.W.N.); 2 Department of Electrical Engineering, Qatar University, Doha 2713, Qatar; mchowdhury@qu.edu.qa

**Keywords:** spiking neural networks, neural encoding, complex exponential neuron, FPGA implementation

## Abstract

This research investigates the implementation of complex-exponential-based neurons in FPGA, which can pave the way for implementing bio-inspired spiking neural networks to compensate for the existing computational constraints in conventional artificial neural networks. The increasing use of extensive neural networks and the complexity of models in handling big data lead to higher power consumption and delays. Hence, finding solutions to reduce computational complexity is crucial for addressing power consumption challenges. The complex exponential form effectively encodes oscillating features like frequency, amplitude, and phase shift, streamlining the demanding calculations typical of conventional artificial neurons through levering the simple phase addition of complex exponential functions. The article implements such a two-neuron and a multi-neuron neural model using the Xilinx System Generator and Vivado Design Suite, employing 8-bit, 16-bit, and 32-bit fixed-point data format representations. The study evaluates the accuracy of the proposed neuron model across different FPGA implementations while also providing a detailed analysis of operating frequency, power consumption, and resource usage for the hardware implementations. BRAM-based Vivado designs outperformed Simulink regarding speed, power, and resource efficiency. Specifically, the Vivado BRAM-based approach supported up to 128 neurons, showcasing optimal LUT and FF resource utilization. Such outcomes accommodate choosing the optimal design procedure for implementing spiking neural networks on FPGAs.

## 1. Introduction

Spiking neural networks (SNNs) represent a promising approach for information encoding, where the firing rate or number of spikes within a certain time period reflects the oscillation properties of biological neurons. In this context, recent research has proposed a mathematical model based on complex exponential neurons [1] that provides a means of encoding and decoding information in artificial neural network systems by leveraging their oscillation features, such as frequency, phase, and amplitude. The model offers a significant advantage over existing approaches as it enables the simplification of complex calculations involving convolution and multiplication to phase addition, which enhances the computational efficiency and performance of artificial neural networks.

The findings of this research are particularly relevant in the context of embedded system design, where there is a need to achieve high precision of neural network output while dealing with limited hardware resources [2,3], such as memory and computational capacity. Thus, this research highlights a promising avenue for improving the performance and efficiency of spiking neural networks, which is a critical factor in enabling their adoption in real-world applications.

### 1.1. Related Work

In this literature review section, we present a comprehensive overview of recent advancements in the field of neural network models based on complex exponential functions. One notable research study introduced the construction of a spiking neural network (SNN) model and its hardware accelerator using FPGA utilizing spiking exponential functions [4]. Another study delved into the implementation of a Multi-Valued Neuron (MVN) model [5], also founded on complex exponential functions, highlighting how MVNs can significantly enhance the functionality of individual neurons. Moreover, a recent investigation focused on MVNs introduced a novel derivative-free convolutional neural network [6] learning algorithm, demonstrating its efficacy in accelerating the learning process while improving generalization capabilities. Remarkably, it is worth noting that, to date, there has been a dearth of hardware accelerators designed to facilitate neural network inference based on these innovative models. The inherent simplicity of derivative-free learning algorithms presents an exciting opportunity to explore the development of dedicated hardware accelerators for neural network learning.

Considerable effort has been devoted to the hardware implementation of spiking neural networks, with a particular focus on FPGA-based and Application-Specific Integrated Circuit (ASIC) systems [7,8]. The paper presented in [9] describes the implementation of a spiking neural network model on a Xilinx FPGA evaluation board, utilizing a hybrid updating algorithm that combines conventional time-stepped updating and event-driven updating techniques, while using 16-bit signed fixed-point number representation. Their model consisted of 16,384 neurons and 16.8 million synapses and achieved an accuracy of 97.06% on the MNIST dataset classification task with a power consumption of only 0.477 W.

Another study [10] presents S2N2, a streaming accelerator for spiking neural networks that efficiently supports axonal and synaptic delays, utilizes binary tensors for addressing events, and achieves a minimum tick-resolution of 30 ns with over three orders of magnitude reduction in input buffer memory utilization.

The study in [11] proposed a holistic optimization framework for encoder, model, and architecture design of FPGA-based neuromorphic hardware, which includes an efficient neural coding scheme, training algorithm, and flexible SNN model represented as a network of IIR filters, achieving state-of-the-art accuracy and outperforming various platforms in terms of latency and throughput.

In [12], a simplified Leaky integrate-and-fire neuron model is used to develop an efficient SNN on Xilinx Virtex 6 FPGA. In a recent study [13], deep convolutional spiking neural networks (DCSNNs) were successfully implemented on low-power FPGA devices, where two backpropagation techniques were compared for object classification tasks. Additionally, another recent work [14] focuses on developing a customizable hardware accelerator for neural network inference models, specifically building a convolutional neural network on an FPGA. This study showcases substantial reductions in power consumption when compared to running the same convolutional neural network model on a laptop processor.

Also, [15] outlines an optimization scheme aimed at improving the performance of the SNN by adjusting biological parameters and then proceeds to implement the SNN on FPGA using the Euler and third-order Runge–Kutta (RK3) methods.

Lastly, implementing complex exponential functions is a crucial part of this project, and it has been developed in many research studies in various ways on FPGA, including polynomial approximation, interpolation, lookup-table-based methods [16], CORDIC IP core [17], two-dimensional interpolation [18], and floating-point implementation [19]. However, these methods often consume a significant amount of FPGA logic cells, such as lookup tables (LUTs) and D flip-flops. Fortunately, most FPGAs have block random access memory (BRAM) [20], which can be utilized to implement complex exponential functions and save considerable FPGA resources. This is particularly important for larger projects like neural network development, where efficient resource utilization is crucial.

In this paper, a complex-exponential-function-based neuron model [1] is designed in MATLAB Simulink using the Xilinx System Generator (SysGen) [21,22] and Xilinx Vivado Design Suite for FPGA implementation on a ZynQ xc7z020clg484-1 FPGA chip, and 8-bit, 16-bit and 32-bit fixed-point number representations are used for the model design.

Xilinx System Generator is an add-on feature for MATLAB Simulink that facilitates using graphical block programming for architecture-level FPGA designs. Xilinx Vivado is an integrated development environment (IDE) [23] for designing digital circuits for Xilinx FPGA and system-on-chip (SoC) devices, supporting various design entry methods, including graphical block diagram entry and HDL code entry.

### 1.2. Outline

The rest of the paper is structured into several key sections. Section 2 offers a mathematical overview of the complex-exponential neuron model, elucidating its foundational principles. Section 3 explores various implementation methods, including BRAM and CORDIC-based designs, realized through Vivado and Simulink, with subsequent analysis. Section 4 presents results and facilitates discussion based on simulations of these implementations. Lastly, Section 5 provides a summary of the research’s core findings and outlines potential future directions, ensuring a logical and coherent flow throughout the paper.

## 2. Background

### 2.1. Mathematical Model

In the realm of complex numbers, a representation is often employed wherein a complex number z=x+iy residing in the complex plane finds its expression as $z=r\cdot e^{i\phi }$ in the phase domain. This representation entails two essential components: the magnitude (*r*), which serves as the vector’s length and is calculated as $r=e^{x}$, and the phase angle (ϕ), signifying the angle between the vector and the x-axis (ϕ=y). Furthermore, Euler’s formula remarkably connects complex exponentials with trigonometric functions, manifesting as $e^{i\pi }+1=0$, with a distinctive instance at ϕ=π, intricately bridging imaginary numbers with the transcendental constants π and *e*. At the core of this representation lie two fundamental equations:

(1) $$|e^{i\phi }|=\sqrt{\cos^{2}\phi +\sin^{2}\phi }=1$$

unveiling the unit magnitude of $e^{i\phi }$, and

(2) $$e^{i\phi }=\cos\phi +i\sin\phi ;\;e^{\bar{i}\phi }=\cos\phi -i\sin\phi$$

illustrating the complex exponential and its complex conjugate. These equations encapsulate the essence of complex number representation and its profound mathematical underpinnings.

In the phase plane, an oscillating neuron E(t) can be written by the complex exponential form as

(3) $$E\left(t\right)=e^{i\omega t+\theta }=e^{\theta }e^{i\omega t}=e^{\theta }\left(\cos\omega t+i\sin\omega t\right)$$

where ω is the angular frequency, and the real component θ is used to make the oscillation amplitude.

The synaptic weight can also be represented by the complex exponential form as $W=e^{i\phi }$, where ϕ represents the phase delay of the neural connection.

Therefore, a weighted input can be represented as

(4) $$E\left(t\right)W=e^{i\omega t+\theta }e^{i\phi }=e^{\theta }e^{i\left(\omega t+\phi \right)}$$

In neural networks, weights act as scaling factors that adjust data as it passes between connected neurons. Input to a neuron is the sum of outputs from previous layer neurons, each weighted by its respective synaptic connection. This process drives information transformation within the network. Pre-synaptic neurons are multiplied by their corresponding weight and then summed up to the post-synaptic neuron. This summation (S) in the post-synaptic neuron can also be represented by a complex exponential form as in Equation (7) [1].

(5)S=E1W1+E2W2(6)=eiω1t+θ1eiϕ1+eiω2t+θ2eiϕ2(7)=eθ1eiω1t+ϕ1+eθ2eiω2t+ϕ2

Three parameters, ω, θ, and ϕ, are used to calculate the weighted sum. This representation aligns with biological plausibility, where the synaptic weight signifies a phase delay introduced to the input neuron’s oscillation, considering minimal signal attenuation over short distances. In biological neural cells, a specialized structure known as myelin plays a vital role in facilitating swift impulse transmission along axons, consequently enhancing the speed of action potential propagation [24]. In this paper, the equation of the complex exponential function is represented by $e^{i\phi }=\cos\phi +i\sin\phi$.

In this analysis, we concentrate on a two-neuron neural network model. Additionally, an in-depth exploration of paper [25], which investigates the complex exponential neuron model and introduces and discusses a three-neuron neural network, including its stability and oscillation conditions.

### 2.2. MATLAB Simulation Report

**Figure 1:**
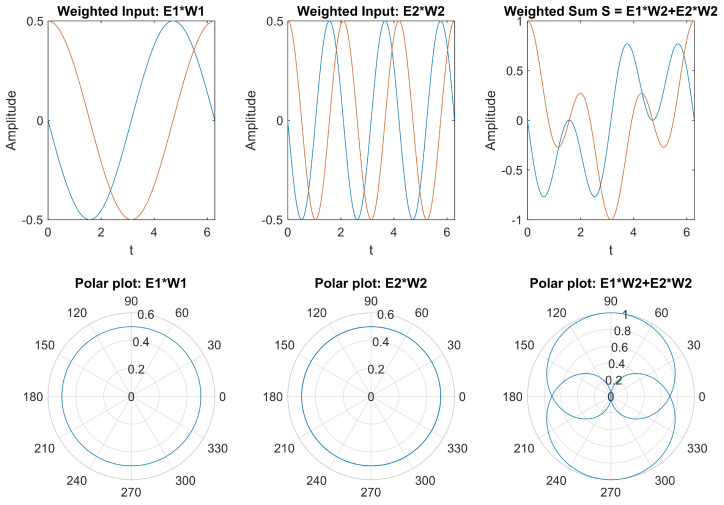
Weighted sum of two complex exponential neurons.

In the MATLAB Simulink, a weighted sum of two neurons model is designed as an example for analyzing the complex exponential neural network. Figure 1 provides a comprehensive depiction of two weighted inputs and their combined weighted sum, plotted individually. The temporal patterns reveal that weighted inputs introduce phase delays to oscillating neurons while preserving their oscillation amplitude. However, the weighted sum continues to oscillate periodically, but its amplitude is no longer constant; instead, it exhibits periodic variations.

**Figure 2:**
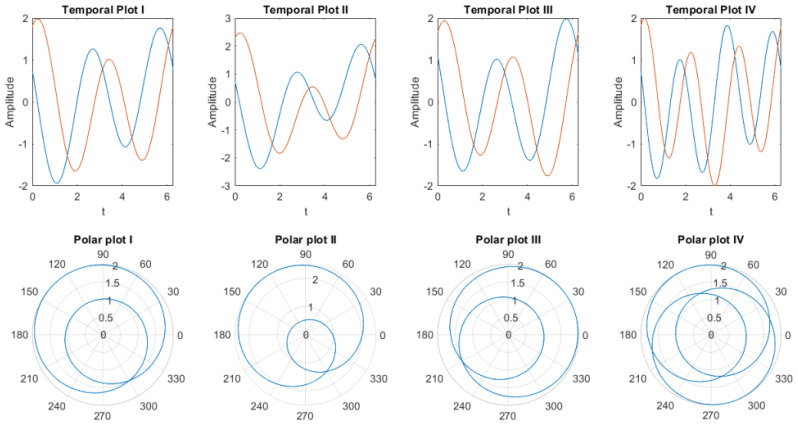
Examples of the weighted sum of two complex exponential neurons with different parameter values.

Figure 2 shows the output of the weighted sum of two neurons E1W1+E2W2 with different parameter values mentioned in the examples below.

- Example 1 in Figure 2 (temporal plot I and polar plot I) shows the output of the weighted sum of two neurons where $\omega_{1}=1,\omega_{2}=2,\theta_{1}=\log\left(0.5\right),\theta_{2}=\log\left(1.5\right),\phi_{1}=\frac{\pi }{2},\phi_{2}=\frac{\pi }{3}$ are used.
- Example 2 (temporal plot II and polar plot II) all parameters of the weighted input of two neurons remain the same except $\theta_{1}=\log\left(1\right)$ is taken. This causes a change in amplitude.
- Example 3, $\theta_{1}$ is changed to $\frac{\pi }{4}$. This changes the phase and orientation of the weighted sum.
- For example, 4 $\omega_{2}$ is set to 3; this dramatically changes the pattern of the weighted sum, which is shown in the polar plot iv.

These four examples are used to demonstrate the effect of different parameters and how changes of one parameters can distinctively change the oscillation patterns of the output. Therefore, any of the parameters can be used to generate unique encoding patterns [26].

## 3. Methodology

### 3.1. Model Design in Simulink and Vivado

In this study, we present four different approaches to implement the weighted sum of two neurons on an FPGA using the Xilinx System Generator (SysGen) of Matlab Simulink and VHDL coding in Vivado Design Suite. The first approach involves utilizing block random access memory (BRAM) HDL blocks to implement the complex exponential function of the weighted sum of two neurons in Matlab Simulink using SysGen. The second approach employs BRAM IP cores and VHDL coding to develop the model in the Vivado Design Suite. In the third approach, CORDIC HDL blocks from the Xilinx toolbox are used in Matlab Simulink to implement the exponential function. Finally, the fourth approach utilizes VHDL coding and CORDIC IP cores to implement the weighted sum of two neurons in the Vivado Design suite.

For each approach, three separate designs were implemented using 8-, 16-, and 32-bit fixed-point data format configurations.

The weighted sum of two neurons requires several input parameters, including $\omega_{1}$, $\omega_{2}$, $\theta_{1}$, $\theta_{2}$, $\phi_{1}$, $\phi_{2}$, and *t*. However, for the sake of simplicity, we kept all input parameters fixed except for *t* across all four approaches. Specifically, we used the following fixed values: $\omega_{1}=1$, $\omega_{2}=2$, $\theta_{1}=\log\left(2\right)$, $\theta_{2}=\log\left(2\right)$, $\phi_{1}=\frac{\pi }{2}$, and $\phi_{2}=\frac{\pi }{2}$. We varied the value of the input parameter *t* within the range of −4 to 4 to cover a complete oscillation cycle.

The outputs of all the approaches were compared with the MATLAB simulation output of Equation (7). The accuracy, latency, and resource utilization of each approach were evaluated and compared to determine the most efficient approach for implementing the weighted sum of two neurons on an FPGA.

Vitis Core Development kit version 2021.1, which includes MATLAB R2021a version 9.10.0.1602886, Xilinx System Generator, and Vivado 2021.1 (64-bit), is used to design, code, and simulate the projects.

#### 3.1.1. BRAM-Based Design (Using SysGen in MATLAB Simulink)

In the FPGA implementation of complex exponential neurons, a crucial task is to implement the function $e^{i\Delta }$. This is achieved by separately implementing the real and imaginary components of the function, which are cosΔ and isinΔ, respectively. Therefore, the real part of the complex exponential output is given by cosΔ, while the imaginary part is given by sinΔ. Here, the Δ is considered as input of the exponential function. In this project, we utilize the periodicity of the cosine and sine functions, which have a period of 2π. Therefore, for any given angle θ, the values of cos(θ) and cos(θ+2π) are the same. The output of the cosine function is a value between −1 and 1, inclusive. To implement the exponential function in BRAM, we consider the input range to be between −3.14 and 3.14 and the output range to be between −1 and 1 since the maximum and minimum value of real (cos(θ)) and imaginary (sin(θ)) output is between 1 and −1. We set the input resolution to a step size of 0.01, resulting in 629 equally spaced values in this range. This approach simplifies the design and reduces the number of memory elements required to implement the exponential function.

**Figure 3:**
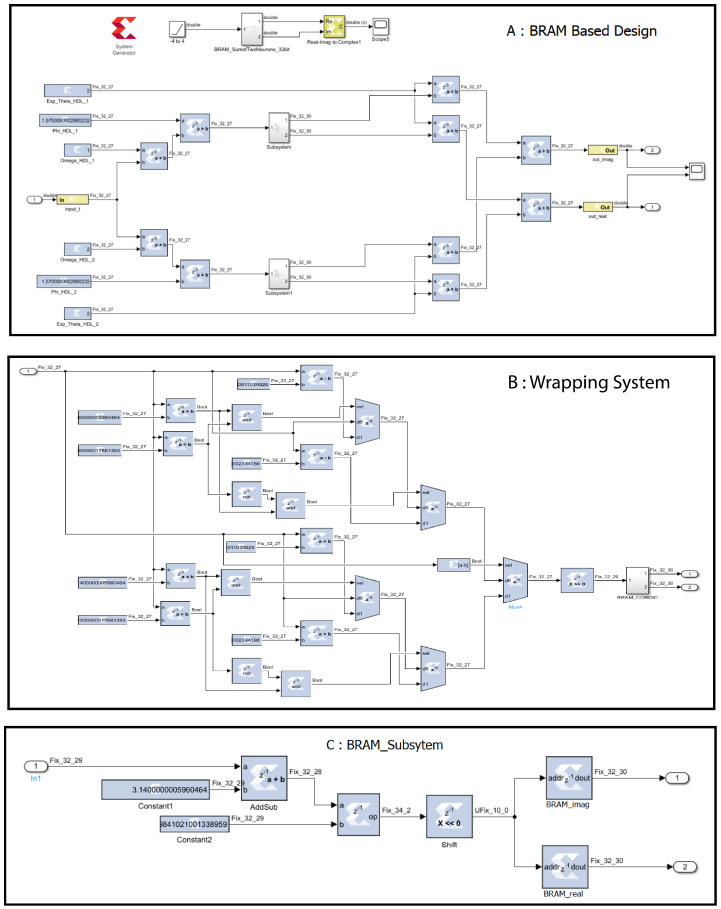
The 32-bit implementation of BRAM-based design in MATLAB Simulink.

In BRAM-based design using the SysGen approach, to implement the complex exponential function, a lookup table with input versus output data of cos and sin is mapped to the block random access memory (BRAM) HDL block in MATLAB Simulink. The block is configured as a fixed-point data format representation. Other graphical HDL blocks, such as the adder, multiplexer, buffer, etc., are used to implement the weighted sum of two complex exponential neurons shown in Figure 3.

Once the design is complete, an IP core is generated using the Xilinx System Generator tool. This IP core is instantiated in Vivado IDE to perform behavioral simulation and generate a hardware implementation report.

#### 3.1.2. BRAM-Based Design in Vivado

In this approach, we utilized the Block Memory Generator tool (version 8.4) of Xilinx LogiCORE to map the block random access memory (BRAM) to implement the complex exponential function in Vivado IDE. To ensure the same level of precision and accuracy, we used a similar lookup table configuration of the BRAM-based design in MATLAB Simulink for the BRAM mapping.

For implementing the weighted sum of two neurons model, we opted for a VHDL coding approach and fixed-point data format to develop the necessary components, including the adder and multiplexer. These components were then mapped to the BRAM to finally complete the implementation.

#### 3.1.3. CORDIC-Based Design (Using SysGen in MATLAB Simulink)

In the CORDIC-based design approach, we take advantage of the built-in Sin_and_Cos function provided by the Xilinx CORDIC 6.0 HDL block in MATLAB Simulink to efficiently implement the complex exponential function. Since the input of the CORDIC block is limited to a range of −π to π, a wrapping subsystem is implemented before the CORDIC IP Core to ensure that the input signal falls within this range. This helps to guarantee the accuracy and precision of the final output.

Once the complex exponential function is obtained, it is combined with other components, such as an adder, multiplexer, and buffer, to implement the weighted sum of two neurons. The fixed-point representation is used throughout the design to maintain precision and reduce the computational overhead.

After finishing the design, an IP core is created using the Xilinx System Generator tool. This IP core is then embedded in Vivado IDE, where behavioral simulation is performed and a hardware implementation report is generated.

#### 3.1.4. CORDIC-Based Design in Vivado

CORDIC-based design in Vivado: In the CORDIC-based design approach in Vivado, we utilize the CORDIC (6.0) IP core of Xilinx to implement the complex exponential function using its built-in Sin and Cos functions and parallel architecture.

**Figure 4:**
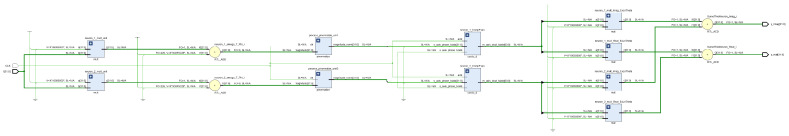
Schematic diagram of 32-bit CORDIC-based design in Vivado.

Similar to the approach in Simulink, we implement a wrapping function, adder, multiplexer, and buffer in VHDL coding to implement the weighted sum of two neurons. Figure 4 shows the schematic diagram of CORDIC-based design in Vivado IDE as an example.

#### 3.1.5. The Fixed-Point Implementation

In the given project, the input range lies between −4 and 4, while the output range is between −1 and 1. To represent the integer part of the input value in binary, only three bits are required as 4 in binary can be represented as 100. One bit is used to represent the sign of the input value, and the remaining bits are reserved for the fractional part.

However, for internal operations such as addition and multiplication, the input values go beyond decimal 16. Therefore, five bits are reserved for the integer part to ensure accurate calculations.

Similarly, for the output values, one bit is used to represent the sign, and the remaining bits are used for the fractional part. Since the maximum and minimum output values are 1 and −1, one bit is reserved for decimal 1, and the remaining bits are used for the fractional part.

By utilizing this fixed-point representation technique, we can allocate more bits to the fractional part, thereby enhancing the precision and accuracy of the final output.

Based on the above conditions, for the Simulink and Vivado-based design, in an 8-bit fixed-point implementation, the input is configured as Fix8_5 data format, with one sign bit, two integer bits, and five fractional bits. The output comes with Fix8_6 data format, with one sign bit, one integer bit, and six fractional bits.

In the 16-bit fixed-point implementation, the input is configured as Fix16_13 data format, with one sign bit, two integer bits and 13 fractional bits. The output comes with Fix16_14 data format, with one sign bit, one integer bit, and 14 fractional bits.

In the 32-bit fixed-point implementation, the input is configured as Fix32_29 data format, with one sign bit, two integer bits, and 29 fractional bits. The output comes with Fix32_30 data format, with one sign bit, one integer bit, and 30 fractional bits.

## 4. Results and Discussion

### 4.1. Simulation Report

#### 4.1.1. 8-Bit Implementation Result

**Figure 5:**
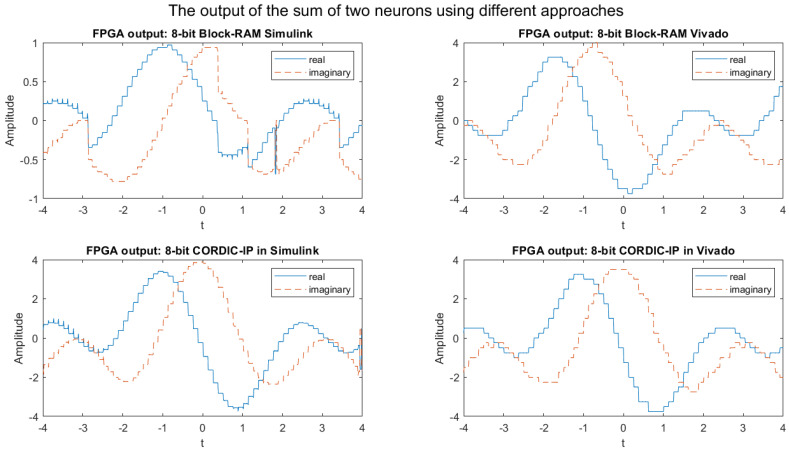
FPGA simulation output graphs of the weighted sum of complex exponential neurons in four different approaches for 8-bit fixed-point implementation.

The graphs presented in Figure 5 depict the output of the FPGA simulation for the weighted sum of complex exponential neurons using four different approaches in the 8-bit fixed-point implementation, which are compared to the output of MATLAB simulation.

**Figure 6:**
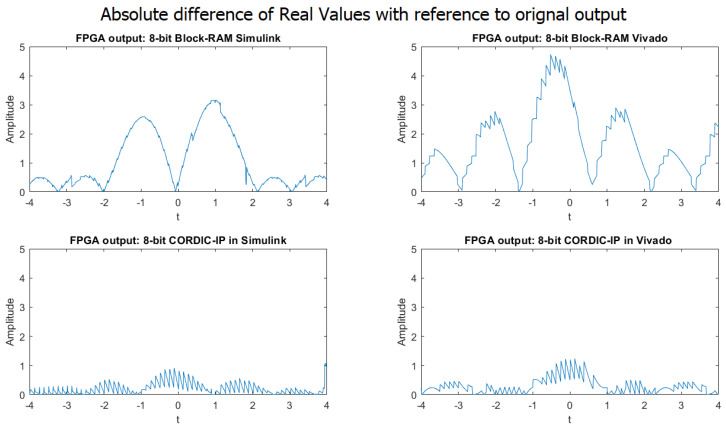
Comparison of absolute difference in real value between MATLAB simulation output and four different approaches in 8-bit implementation.

The graphs in Figure 6 show the absolute difference in the real values of the weighted sum of complex exponential neurons using BRAM-based and CORDIC-based designs in both Simulink and Vivado for the 8-bit fixed-point implementation.

It was observed that while the shape of the output signals produced by the four different approaches were similar to the output produced by MATLAB, the precision of the outputs was very poor when compared to the expected output generated by MATLAB.

**Figure 7:**
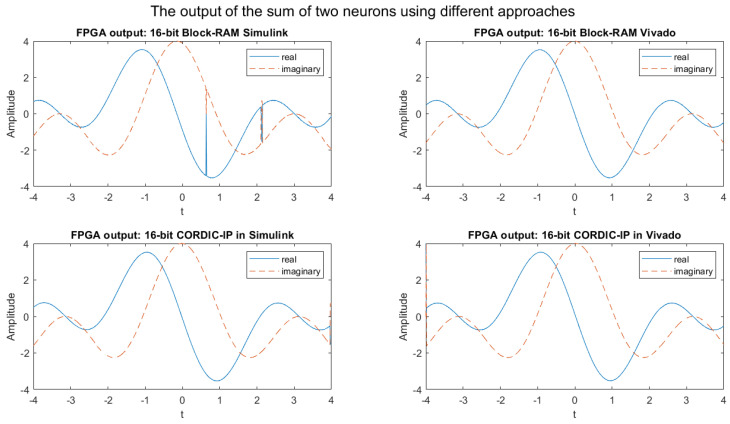
FPGA simulation output graphs of the weighted sum of complex exponential neurons in four different approaches for 16-bit fixed-point implementation.

As the MATLAB simulation output for the weighted sum of two neurons in the 16-bit implementation is comparable to the output displayed in Figure 7, we have excluded the graph for the MATLAB simulation output of the weighted sum of two neurons.

#### 4.1.2. 16-Bit Implementation Result

**Figure 8:**
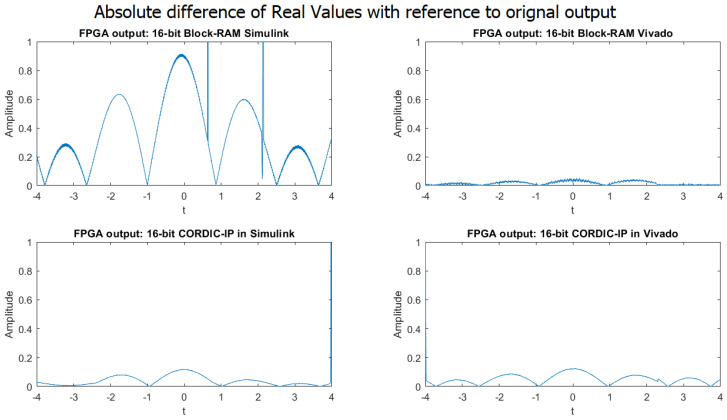
Comparison of absolute difference in real value output between MATLAB simulation and four different approaches in 16-bit implementation.

The FPGA simulation outputs for the weighted sum of complex exponential neurons using four different approaches in 16-bit fixed-point implementation are presented in Figure 7. Figure 8 displays the absolute difference of real values of the weighted sum of two complex exponential neurons for the same four approaches in 16-bit fixed-point implementation. The comparison of Figure 7 and Figure 8 indicates that the outputs of the weighted sum of two neurons in 16-bit fixed-point implementation for all four approaches are significantly more accurate and closely matched to the output of the MATLAB simulation.

From the Figure 5 and Figure 7, we can observe some glitches in the output of the BRAM Simulink-based designs; this might be because of the overflow in the calculation in the wrapping system design. We will fix it in our future research.

#### 4.1.3. 32-Bit Implementation Result

**Figure 9:**
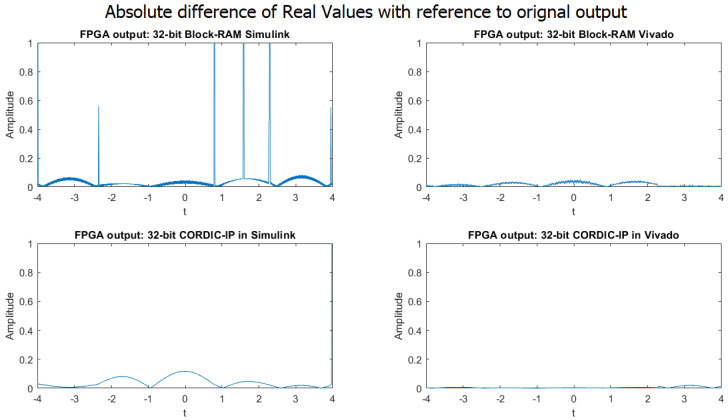
Comparison of absolute difference in real value between MATLAB simulation output and four different approaches in 32-bit implementation.

By examining the graphs in Figure 9 in the 32-bit implementation presented in the paper, it is evident that the absolute difference between the output generated by the MATLAB simulation and the output produced by the four different design approaches is significantly lower compared to the 16-bit implementation. This implies that the accuracy and precision of the output are greatly improved by using 32-bit implementation.

#### 4.1.4. MAE of the Four Different Implementation Approaches

**Figure 10:**
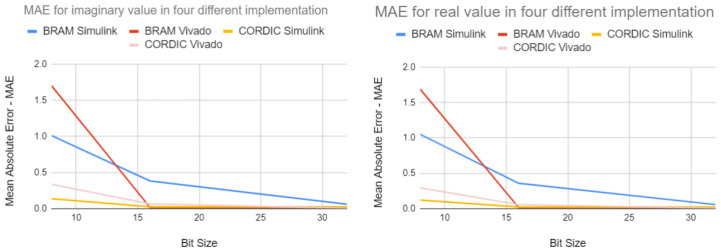
Mean Average Error of the output of four different implementation approaches for real and imaginary values.

The Mean Average Error graphs in Figure 10 clearly indicate that as the bit size increases, the Mean Average Error becomes minimized for all four implementation approaches. This suggests that the outputs generated by these implementations are becoming more precise and closer to the expected output. Though it is shown in the report that MAE has been calculated using the output of the real value, a similar result is found for the output of the imaginary value.

#### 4.1.5. Discussion Summary

The discussed results focus on the performance of different fixed-point implementation approaches of complex exponential neurons in FPGA simulations. The paper compares the output of the FPGA simulations with that of MATLAB simulations. The results show that the 8-bit fixed-point implementation has poor precision compared to the 16-bit and 32-bit implementations. The 16-bit implementation outputs are more accurate and closely matched to the MATLAB simulation outputs. The 32-bit implementation further improves the accuracy and precision of the output, as evident by the significantly lower absolute difference between the output generated by the MATLAB simulation and the output produced by the four different design approaches. The Mean Average Error graphs demonstrate that the accuracy and precision of the output increase as the bit size increases, minimizing the Mean Average Error for all four implementation approaches. Overall, the results suggest that the use of higher bit sizes in fixed-point implementation approaches can improve the accuracy and precision of the output, making them more reliable and useful in practical applications.

### 4.2. Hardware Implementation Report

The field of FPGA hardware design offers multiple approaches for implementing a design, each with its own set of advantages and disadvantages. This article will focus on comparing four design methods: BRAM-based design in both Simulink and Vivado, and the CORDIC IP-based design in both Simulink and Vivado for 8-, 16-, and 32-bit implementation. The comparison between these methods will be based on their speed, power requirements, and resource usage.

The BRAM-based implementation uses block RAMs as the primary storage element for the design. The advantage of this approach is that it enables the implementation of designs with high memory requirements, and it can be optimized for power and area. However, this approach may not be optimal for designs with high operating frequencies since the latency of accessing the block RAM can be high, leading to a reduced operating frequency.

The CORDIC IP-core-based implementation involves using pre-designed and pre-verified blocks of digital circuits (IP cores) that can be integrated into a larger design. This approach can reduce development time and simplify the design process. Additionally, IP cores are typically optimized for performance, which can result in high operating frequencies.

This project has utilized the ZYNQ-7 ZC702 Evaluation Board library in Vivado for implementing all the different methods. The board used in this project has a part name of xc7z020clg484-1, and it offers 484 I/O pins, LUTs of 53,200, FFs of 106,400, and DSPs of 220, among other resources.

#### 4.2.1. WNS Report

**Figure 11:**
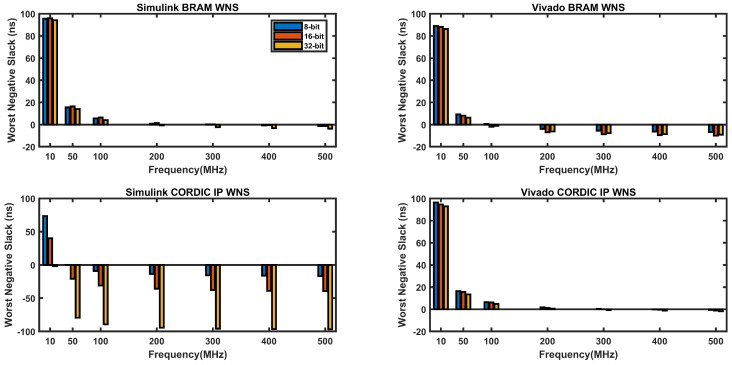
WNS vs. operating frequency graphs in four different approaches.

Figure 11 shows the worst negative slack (WNS) in nanoseconds for different clock frequencies for four different types of implementations: BRAM-based in Simulink, BRAM-based in Vivado, CORDIC-based in Simulink, and CORIDC-based in Vivado, with different data widths of 8 bits, 16 bits, and 32 bits.

WNS represents the amount of time by which the path with the worst timing violates the clock period, which means that the design fails to meet timing at that frequency. A negative WNS means that the design is failing timing, while a positive WNS means that the design is meeting timing. Ideally, a positive value of nearly zero or zero is considered as a good design for a particular clock frequency.

From Figure 11, it appears that Vivado BRAM and Vivado CORDIC designs have better worst negative slack (WNS) values than Simulink BRAM and Simulink CORDIC designs for all operating frequencies. This means that Vivado designs are meeting timing more easily compared to Simulink designs in the selected frequency range (10 Mzh to 500 MHz).

Furthermore, it can be observed that as the operating frequency increases, the WNS values become more negative for all designs. This is expected since higher frequencies lead to shorter clock cycles, which gives less time for the signals to propagate through the circuit. As a result, it becomes harder for the designs to meet timing at higher frequencies.

In terms of the design types, it appears that the CORDIC designs have worse WNS values compared to the BRAM designs for all operating frequencies.

It can also be observed that bit size has less significance for all designs except the CORDIC-based design in Simulink, where the increase in bit size leads to a decrease in the WNS value.

Overall, the WNS values provide an indication of the timing performance of the designs, but other factors such as resource usage and power consumption also need to be considered when comparing different design implementations.

#### 4.2.2. Max Operating Frequency

Maximum Operating Frequency is calculated by the formula below:

(8) $$\begin{array}{c} f_{max}=\frac{1}{T_{S}-WNS} \end{array}$$

where $f_{max}$ is the maximum clock frequency, WNS is worst negative slack, $T_{S}$ is the minimum time required to complete a sequence. In maximum operating frequency, WNS will be nearly 0 and $f_{max}=\frac{1}{T_{S}}$

**Figure 12:**
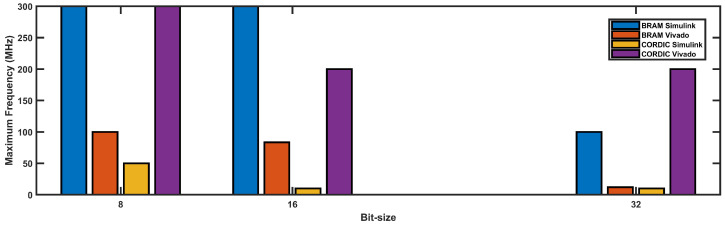
Maximum operating frequency vs. bit-size graph for different implementation approaches.

If we look at Figure 12, it can be observed that the maximum frequency achieved varies for different bit sizes and different implementations. For the BRAM-based implementations, the Simulink implementation achieves a higher maximum frequency compared to the Vivado implementation for all bit sizes. On the other hand, for the CORDIC-based implementations, both the Simulink and Vivado implementations achieve the same maximum frequency of 300 MHz for 8-bit and 16-bit implementations, while for 32-bit implementation, the Vivado implementation achieves a higher maximum frequency of 200 MHz compared to the Simulink implementation.

Overall, the BRAM-based implementations achieve higher maximum frequencies compared to the CORDIC-based implementations. Moreover, increasing the bit-size results in a decrease in the maximum frequency achieved for all implementations, except for the Vivado CORDIC implementation for 32-bit, where it achieves a higher maximum frequency compared to the 16-bit implementation.

#### 4.2.3. Resource Usages

Table 1 shows the usage of lookup tables (LUTs) and flip-flops (FFs) for different implementations using different design tools. For the BRAM-based designs, the LUT usage is comparatively low, ranging from 41 to 337 for Vivado and from 286 to 2904 for Simulink, across all bit sizes. The FF usage for the BRAM-based designs is also low, ranging from 0 to 116 for Vivado and from 514 to 5336 for Simulink. On the other hand, the CORDIC-based designs have a higher LUT and FF usage, especially for the higher bit sizes. The LUT usage for the CORDIC-based designs ranges from 612 to 7660 for Simulink and from 548 to 7473 for Vivado. The FF usage for the CORDIC-based designs ranges from 196 to 874 for Simulink and from 516 to 7217 for Vivado.

Overall, it can be observed that the BRAM-based designs require relatively fewer FPGA resources in terms of LUT and FF usage compared to CORDIC-based designs. However, as the bit size increases, the resources required for CORDIC-based designs increase significantly. It is also interesting to note that the usage of FPGA resources is different for different design tools, with Simulink generally requiring more resources than Vivado. These findings can be useful for selecting the appropriate implementation method based on the available FPGA resources and the desired performance requirements.

**Figure 13:**
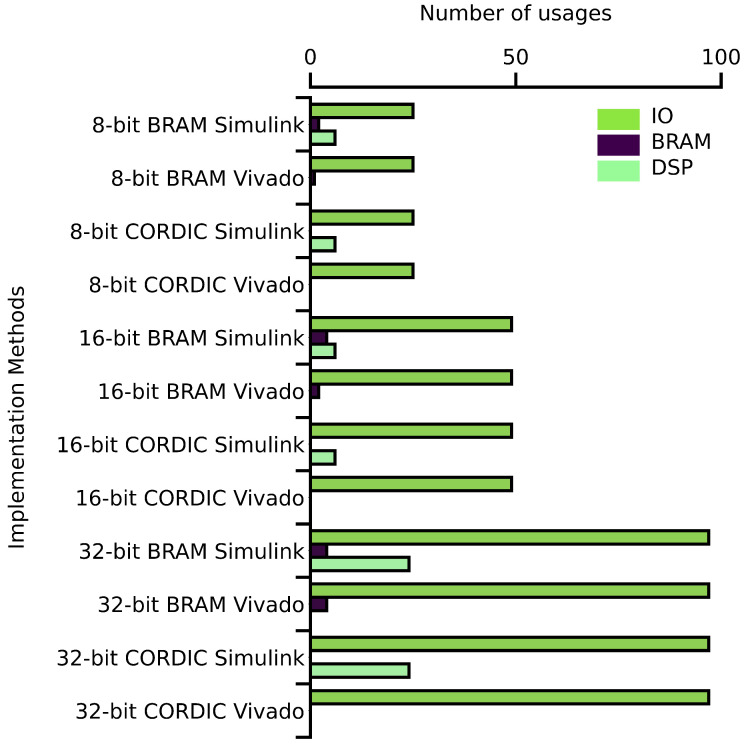
Other resource utilization graph.

Upon analyzing Figure 13, it is evident that as the bit size increases in each implementation method, the IO usage also increases. Additionally, it can be observed that BRAMs are only utilized in the BRAM-based implementations while the DSP units are only used in the Simulink-based implementations. Interestingly, no DSP unit is utilized in the Vivado-based implementation. Overall, the usage of IO, BRAM, and DSP units vary depending on the implementation method and the bit size used.

#### 4.2.4. Power Requirement

**Figure 14:**
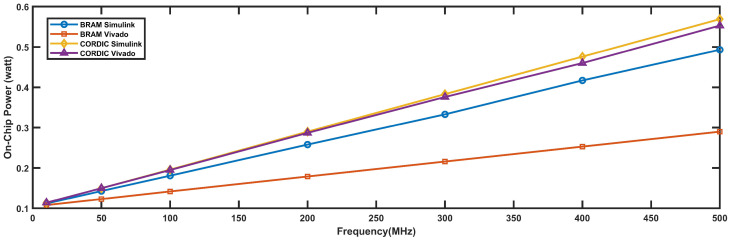
Frequency vs. power requirement graph for 16-bit implementation.

The power requirements in watts for four different types of 16-bit FPGA implementations, namely BRAM Simulink, BRAM Vivado, CORDIC Simulink, and CORDIC Vivado, for different operating frequencies are shown in Figure 14. It can be observed that as the operating frequency increases, the power requirement for all four implementations also increases. Among the four implementations, BRAM Simulink requires the least amount of power while CORDIC Simulink and CORDIC Vivado require the highest amount of power. At a frequency of 10 MHz, the power requirement for all four implementations is quite close to each other, ranging from 0.108 watts to 0.114 watts. However, at a frequency of 500 MHz, the power requirement varies significantly among the implementations, ranging from 0.29 watts for CORDIC Simulink to 0.553 watts for CORDIC Vivado. Therefore, it is important to consider the power requirement of different FPGA implementations while selecting the appropriate design for a particular application, especially if the operating frequency is high.

#### 4.2.5. Discussion Summary

In this hardware implementation report, four different design methods were compared for implementing a two-neuron network on an FPGA, with a focus on speed, power requirements, and resource usage. The BRAM-based implementation in Vivado achieved better WNS values and higher maximum frequencies compared to the Simulink implementation, while CORDIC-based designs had worse WNS values due to the algorithm’s complexity. BRAM-based designs had comparatively lower resource usage, with Vivado implementation requiring fewer resources compared to Simulink.

### 4.3. Hardware Implementation Report for the Design with All the Input Variables in Use

**Figure 15:**
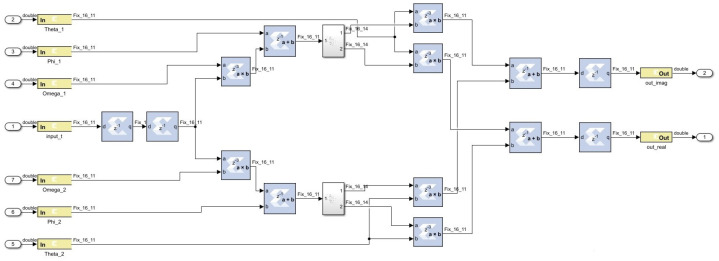
16-bit implementation of BRAM-based design in MATLAB Simulink with all the input variables that were actively employed in the FPGA’s implementation.

In the initial stages of our design, the computation of the weighted sum for a two-neuron model within the FPGA was driven by a single dynamic input, the “time” (t) variable, while other critical parameters—specifically ω, θ, and ϕ—were set as constants, merely occupying ROM space. This configuration did not accurately represent the FPGA’s resource usage when handling multiple dynamic inputs. To rectify this and simulate a more realistic resource consumption scenario, we expanded our study to include all variables as inputs, one example is shown in the Figure 15. Employing four distinct implementation strategies, we leveraged the robust ZYNQ-7 ZC706 Evaluation Board, which is powered by the xc7z045ffg900-2 FPGA part. The new board was chosen for its ample I/O pins, which were necessary to meet our project’s expanded requirements. This board boasts an array of features, such as 362 usable I/O pins, 218,600 lookup tables (LUTs), 437,200 flip-flops (FFs), 545 block RAMs (BRAMs), and 900 digital signal processors (DSPs), to name a few.

**Figure 16:**
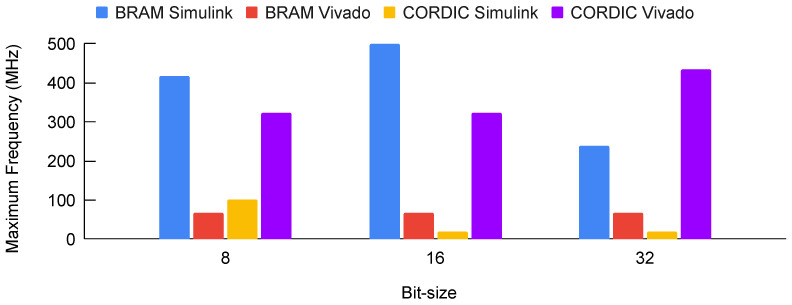
Maximum operating frequency vs. bit-size graph for different implementation approaches when all input variables were actively employed in the FPGA’s implementation.

Figure 16 illustrates the peak operating frequencies attained through various implementation methods across differing bit sizes. An analysis of Figure 12 and Figure 16 (which depict scenarios with a singular dynamic input (time t) and other variables held constant) reveals a similar pattern in maximum operating frequency achieved across the four implementation methods. Intriguingly, when all input variables were actively employed in the FPGA’s implementation of the two-neuron model’s weighted sum, certain methods demonstrated an ability to reach elevated maximum operating frequencies. This indicates the potential for improved performance when the model is fully parameterized with dynamic inputs.

**Figure 17:**
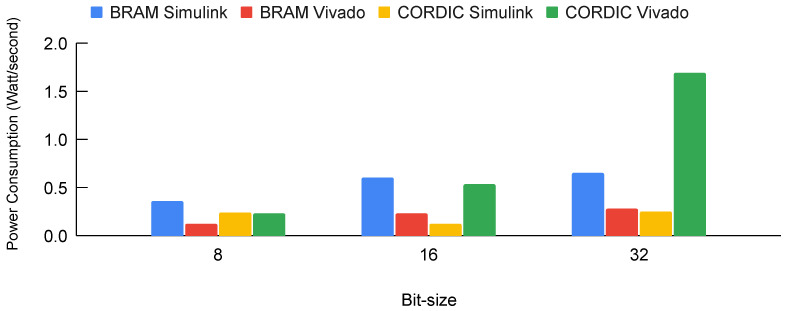
Power consumption across four different implementation methods when all input variables were used in FPGA implementation.

The power consumption across four distinct implementation methods, as observed when all input variables were dynamically integrated into the FPGA’s framework, is comprehensively illustrated in Figure 17.

Table 2 presents a detailed overview of resource utilization for various implementations, highlighting the consumption metrics when all input variables were incorporated into the FPGA implementation. Similar to the implementations with a single variable input, the CORDIC Vivado model, in these multi-variable input scenarios, continues to show the highest resource consumption compared to other methods. Conversely, the BRAM Vivado model maintains its status as the most resource-efficient approach.

Overall, integrating all input variables into the FPGA implementation, while leading to an increase in power consumption and resource utilization, simultaneously enables a significant boost in operating frequency. This enhancement was consistently observed across different implementation methods, regardless of whether they used all input variables or not, indicating a uniform trend in implementation results.

### 4.4. Multi-Neuron Implementation Report

After successfully implementing the weighted sum two neurons model, our objective was to evaluate the capabilities of four different implementation methods for real-time computing of the weighted sum of N number of inputs or neurons, where N is 4, 8, 16, 32, 64, 128. To achieve this, we utilized the ZYNQ-7 ZC702 Evaluation Board and measured how many neurons could be successfully implemented on the FPGA board and how much of the resources each of the four implementation methods consumed. To achieve real-time computing of the weighted sum of N inputs, we used parallel instantiation of the sum of the two-neuron model. We only considered 16-bit and 32-bit implementations, taking into account output precision.

Table 3 shows the maximum number of neurons achieved by different implementation methods on the ZYNQ-7 ZC702 Evaluation Board for both 16-bit and 32-bit implementations. For the 16-bit implementation, Simulink BRAM achieved a maximum of 64 neurons, while Simulink CORDIC and Vivado CORDIC achieved only 32 neurons each. BRAM-based method in Vivado achieved the highest number of neurons among all the methods with 128 neurons.

For the 32-bit implementation, the maximum number of neurons achieved is significantly lower than the 16-bit implementation. Simulink BRAM achieved only 16 neurons, while Simulink CORDIC and Vivado CORDIC achieved only 8 neurons each. Vivado BRAM, again, achieved the highest number of neurons with 64. The data show that the number of neurons that can be successfully implemented on the board varies significantly based on the implementation method and the bit size used.

**Figure 18:**
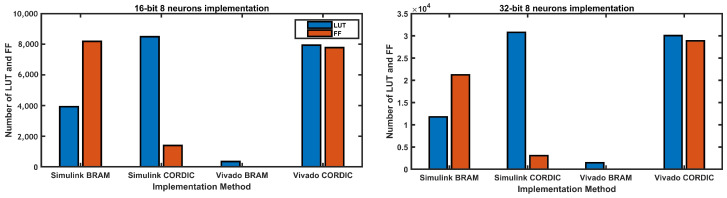
LUT and FF usage in 16-bit and 32-bit implementations for the weighted sum of eight neurons.

Figure 18 displays the LUT and FF resource utilization of the weighted sum of eight neurons for four different implementation methods using a 16-bit and 32-bit implementation. In the case of the 16-bit implementation, the Simulink BRAM and Vivado BRAM implementations consumed the lowest LUT and FF resources, with the latter not utilizing any FF resources. On the other hand, the Simulink CORDIC and Vivado CORDIC implementations consumed significantly higher LUT and FF resources than the BRAM implementations. For the 32-bit implementation, the BRAM implementations (Simulink BRAM and Vivado BRAM) consumed fewer LUT and FF resources than the CORDIC implementations (Simulink CORDIC and Vivado CORDIC), with Vivado CORDIC consuming the most LUT and FF resources. The utilization of LUT and FF resources in the weighted sum of eight neurons implementation was considerably less for the BRAM-based design in Vivado for both 16-bit and 32-bit implementations when compared to other methods.

From Table 4, it can be observed that the highest frequency achieved in the eight-neuron implementation for both 16-bit and 32-bit designs was with Vivado CORDIC method at 200 MHz. The Simulink BRAM and Vivado BRAM implementations achieved the lowest frequencies, with 250 MHz and 50 MHz for 16-bit and 53 MHz for 32-bit. The Simulink CORDIC implementation had the lowest frequency, achieving only 19 MHz and 9 MHz for 16-bit and 32-bit, respectively.

## 5. Conclusions and Future Work

In this paper, different design methods were compared for implementing a two-neuron network and a multi-neuron network on an FPGA. The comparison was based on speed, power requirements, and resource usage. The results showed that the BRAM-based implementation in Vivado achieved better WNS values and higher maximum frequencies compared to the Simulink implementation. BRAM-based designs had comparatively lower resource usage, with the Vivado implementation requiring fewer resources compared to Simulink.

For the multi-neuron implementation, the number of neurons that could be successfully implemented on the board varied significantly based on the implementation method and the bit-size used. The Vivado BRAM-based design achieved the highest number of neurons among all the methods with 128 neurons. The utilization of LUT and FF resources in the multi-neuron implementation was considerably less for the BRAM-based design in Vivado for both 16-bit and 32-bit implementations when compared to other methods.

Overall, the results show that the choice of implementation method can significantly impact the performance and resource usage of the FPGA design. Therefore, it is important to carefully evaluate different design options and select the one that best meets the specific requirements of the application.

In order to advance the research in this field, the next step would be to create a fully functional complex exponential neural network model. The current work has mainly concentrated on the information encoding aspect of the neural network model. However, in order to develop a complete model, further research is required to establish the decoding method and create a neural network model using complex exponential neurons. Additionally, it would be important to compare the performance of the complex exponential neural network model with other existing neural network models to assess its effectiveness.

## Figures and Tables

**Table 1:** LUT and FF usages for different implementations.

Resource Usages	Bit Size	BRAM Simulink	BRAM Vivado	CORDIC Simulink	CORDIC Vivado
LUT usages	8	286	41	612	548
LUT usages	16	965	202	2160	2094
LUT usages	32	2904	337	7660	7473
FF usages	8	514	0	196	516
FF usages	16	2190	0	412	1944
FF usages	32	5336	116	874	7217

**Table 2:** FPGA resource usages for different implementations when using all input variables in FPGA implementation.

Resource Usages	Bit Size	BRAM Simulink	BRAM Vivado	CORDIC Simulink	CORDIC Vivado
LUT	8	296	341	622	835
LUT	16	983	234	2116	2148
LUT	32	2940	639	7694	7777
FF	8	546	20	196	516
FF	16	1966	0	508	1944
FF	32	5336	88	874	7218
BRAM	8	2	1	0	0
BRAM	16	4	2	0	0
BRAM	32	4	4	0	0
DSP	8	6	0	6	0
DSP	16	6	6	6	6
DSP	32	24	24	24	24
IO	8	73	73	73	73
IO	16	145	145	145	145
IO	32	289	289	289	289

**Table 3:** Maximum number of neurons successfully implemented by the four implementation methods.

8-Neuron Method	Max. Number of Neurons	
	16 Bit	32 Bit
Simulink BRAM	64	16
Simulink CORDIC	32	8
Vivado BRAM	128	64
Vivado CORDIC	32	8

**Table 4:** Maximum frequency achieved by different implementation methods.

8-Neuron Method	Maximum Frequency (MHz)	
	16 Bit	32 Bit
Simulink BRAM	250	150
Simulink CORDIC	19	9
Vivado BRAM	50	53
Vivado CORDIC	200	200

## Data Availability

Data are contained within the article.

## Abbreviations

The following abbreviations are used in this manuscript:

FPGA	Field-Programmable Gate Array
ASIC	Application-Specific Integrated Circuit
DSP	Digital Signal Processor
HDL	Hardware Description Language
VHDL	VHSIC Hardware Description Language
LUT	Lookup Table
FF	Flip-Flop
SSN	Simultaneous Switching Noise
BRAM	Block RAM (Random Access Memory)
CORDIC	Coordinate Rotation Digital Computer
IO	Input/Output
IP	Intellectual Property
